# Interferon-stimulated gene 15 facilitates BTV replication through interacting with the NS1 protein

**DOI:** 10.3389/fmicb.2023.1212242

**Published:** 2023-08-11

**Authors:** Di Kang, Guorui Zhang, Zhonghui Zhang, Zhancheng Tian, Shandian Gao, Guangyuan Liu, Guiquan Guan, Jianxun Luo, Hong Yin, Junzheng Du

**Affiliations:** ^1^State Key Laboratory for Animal Disease Control and Prevention, College of Veterinary Medicine, Lanzhou University, Lanzhou Veterinary Research Institute, Chinese Academy of Agricultural Sciences, Lanzhou, China; ^2^College of Veterinary Medicine, Gansu Agricultural University, Lanzhou, China; ^3^Jiangsu Co-innovation Center for Prevention and Control of Important Animal Infectious Diseases and Zoonoses, Yangzhou University, Yangzhou, China

**Keywords:** BTV, NS1 protein, ISG15, virus replication, ISGylation

## Abstract

Bluetongue virus (BTV) infection effectively activates the innate immune response, followed by the expression of interferon (IFN) and multiple interferon-stimulated genes (ISGs). ISG15 is one of the most induced ISGs, and often plays a role in inhibiting virus replication. This study aims to explore the role and specific mechanisms of ovine ISG15 (oISG15) in BTV infection. We found that the transcription level of oISG15 was upregulated in a time-dependent and BTV multiplicity of infection-dependent manner. The overexpression of exogenous oISG15 enhances BTV replication, whereas the knockdown of endogenous oISG15 inhibits BTV replication. The viral protein in wild-type oISG15-overexpressed cells and ISGylation defective oISG15-overexpressed cells have no significant differences, which indicated that oISG15 promoted BTV replication in an ISGylation-independent manner. A co-immunoprecipitation assay showed that four viral BTV proteins—VP3, VP4, VP5, and NS1—interacted with oISG15. We also found that the VP4 and NS1 proteins associated with ubiquitin via co-immunoprecipitation, and that oISG15 overexpression improved the stability of both proteins. Further results showed that the degradation of NS1 was involved in lysine 63-linked polyubiquitin. This suggested that oISG15 may interfere with NS1 degradation via the autophagy pathway. This study provides new insights on the interaction between BTV and ISG15, and enriches our understanding of the regulation and biological function of ISG15 with virus replication.

## 1. Introduction

Bluetongue (BT) is an infectious, non-contagious disease of ruminants caused by bluetongue virus (BTV). The mortality of BT could reach to 50–70% in infected sheep flocks, resulting in huge economic losses around the world (Mayo et al., 2017). BTV is mainly transmitted by biting midges such as *Culicoides* spp., and the distribution of BT from tropical to temperate zones coincides with vector insect species (Belbis et al., 2017). BTV belongs to the genus *Orbivirus* in the family Reoviridae. The BTV genome is composed of 10-segmented double-stranded RNA (dsRNA) and encodes seven structural (VP1–VP7) and six non-structural proteins (NS1–NS3, NS3A, NS4, and NS5). The viral genome is surrounded by VP1 (RNA-dependent RNA polymerase), VP4 (capping enzyme), and VP6 (viral helicase), which constitute the replication complex (Patel and Roy, 2014). The VP3 and VP7 trimers are encapsulated on this complex and form the core particle. The outer layer consists of the most variable viral proteins—VP2 and VP5. This layer is responsible for cell attachment and entry into the cells (Roy et al., 2009). The non-structural protein NS1 forms viral tubules in BTV-infected cells to improve the translation efficiency of viral proteins (Boyce et al., 2012). NS2 is necessary for the formation of viral inclusion bodies and recruits proteins for viral assembly (Kar et al., 2007). NS3 and NS3A were reported to suppress the transcription of interferon β (IFN-β) and participate in virus trafficking and egress (Celma and Roy, 2009; Chauveau et al., 2013). NS4 was found to downregulate the translation of type I IFN and interferon-stimulated genes (ISGs) (Ratinier et al., 2016). The NS5 protein was newly detected in BTV-infected BSR cells. It responds to the degradation of ribosomal RNAs during infection and reduces host-cell protein synthesis (Mohd Jaafar et al., 2023).

After BTV infection, the pathogen-associated molecular patterns (PAMPs) of BTV, especially genomes, can be recognized by the pattern recognition receptors (PRRs) of host cells. For instance, TLR3, RIG-I, MDA5, PKR, and TRIF-dependent DexD/H-box helicases were found to be responsible for BTV infection (Chauveau et al., 2012). The multiple subtypes of IFN-I (IFN-α/β/ω) are synthesized after the activation of these receptors and several signaling cascades. This process is critical for the response to innate immunity following BTV infection (Rodriguez-Calvo et al., 2014). Subsequently, hundreds of ISGs are transcribed and induced by the IFN-I signal pathway (Vitour et al., 2015). ISG15 was one of the most significantly differentially expressed ISGs after BTV infection in sheep testicular cells (Du et al., 2016). The ISG15 precursor is cleaved by the protease to form the mature molecular, which is characterized by the LRLRGG motif at the carboxy terminal. The target proteins covalently conjugate onto ISG15 through this motif (Speer et al., 2016). ISG15 conjugation (ISGylation) requires a three-step enzymatic process that involves an E1 activating enzyme, an E2 conjugating enzyme, and an E3 ligase, which are all strongly induced by IFN-I. ISGylation can be reversed by the ubiquitin specific protease USP18 (Dzimianski et al., 2019). As an interferon-induced protein, either by directly interacting with viral proteins or regulating the host cells’ pathway, ISG15 often plays an antiviral role in the process of virus infection. This is mostly completed through ISGylation modification. For instance, Tsg101 belongs to the cellular endosomal sorting complex required for transport (ESCRT) and responses to the vacuolar sorting machinery, where it helps select cargo for incorporation into vesicles that bud into multivesicular bodies (MVBs). ISG15 can modify Tsg101 in IAV-infected cells, thus limiting the transportation of IAV hemagglutinin (HA) to the cell surface and virus release (Pincetic and Leis, 2009; Sanyal et al., 2013). Several studies have found that ISGylation enhances the IFN signal pathway after the infection of hepatitis C virus (HCV), Japanese encephalitis virus (JEV), and Sindbis virus to suppress virus replication (Hsiao et al., 2010; Shi et al., 2010; Ganesan et al., 2016). However, it was reported that the ISGylation of RIG-I leads to autophagic degradation and negatively modulates the innate immune response (Kim et al., 2008). In addition, ISG15 exists as a free intracellular protein that can also secrete extracellularly to regulate the proliferation and capabilities of natural killer cells (D’Cunha et al., 1996).

Although the structure and assembly of BTV have been well characterized as the model of *Orbivirus*, the interaction between BTV and ISG is poorly understood. In this study, we found that ovine ISG15 (oISG15) enhances BTV replication independent of ISGylation, and that the knockdown of endogenous ISG15 inhibits BTV replication. The abundance of viral proteins interacting with oISG15 was found to increase following the overexpression of oISG15. Meanwhile, oISG15 could enhance the stability of VP4 and NS1, both of which were modified by ubiquitin. Further data suggested that oISG15 may disturb the degradation of the lysine 63 (K63)-linked polyubiquitin of NS1. This study provides new insights into the interaction between BTV and oISG15.

## 2. Materials and methods

### 2.1. Cells and viruses

Ovine-derived kidney cells (MDOK, ATCC-CRL-1633) and HEK-293T cells were cultured at 37°C in Dulbecco’s modified Eagle’s medium (DMEM, Gibco) containing 10% fetal bovine serum (FBS, Gibco). Baby hamster kidney 21 cells (BHK-21, ATCC-CCL-10) were cultured at 37°C in modified Eagle’s medium (MEM, Hyclone) supplemented with 5% FBS. MDOK, HEK-293T, and BHK-21 cells were preserved in our lab. The BTV-1 strain (GS/11) was propagated in BHK-21 cells, and the virus titer was determined with plaque formation assay.

### 2.2. Virus infection and cloning of oISG15 gene

A monolayer of MDOK cells was seeded in 12-well plates with approximately 80–90% confluency and inoculated with BTV-1 at a multiplicity of infection (MOI) of 1. After 1 h of adsorption, the cells were maintained in DMEM supplemented with 2% FBS and collected at 12, 24, and 48 h post-infection (hpi). The other group of MDOK cells was infected with BTV at MOIs of 0, 0.1, 1, and 10 for 48 h using the same method as mentioned above. The infected cells were washed with cold PBS three times, and the total RNA was isolated using TRIzol reagent (Invitrogen). The cDNA of total RNA was synthesized using a PrimeScript™ RT reagent kit with gDNA Eraser (TaKaRa). The oISG15 mRNA level was determined using real-time qRT-PCR, and the primer sequences are presented in Table 1.

**TABLE 1:** PCR primers and siRNA sequences used in this study.

Primer	Sequence of oligo nucleotide (5′-3′)	Purpose
oISG15-F	ATGGGCGGGGACCTGAAGGTGA	Gene cloning
oISG15-R	CTACCCACCCCGCAGACGTAGAT	
oISG15-HA-F	CGGGGTACCGGCGGGGACCTGAAGGTGAAG	Construction of oISG15-HA
oISG15-HA-R	CCGCTCGAGTCACCCACCCCGCAGACGTAG	
mut-oISG15-HA-F	CGGGGTACCGGCGGGGACCTGAAGGTGAAG	Construction of mut-oISG15-HA
mut-oISG15-HA-R	CCGCTCGAGCTACGCAGCCCGCAGACGTAG	
qoISG15-F	TGCTCTGCTCACCCCAACT	qRT-PCR
qoISG15-R	CCGTTCCCCTTTTGCTCTC	
qβ-actin-F	CCCTGGAGAAGAGCTACGAG	qRT-PCR
qβ-actin-R	GAAGGAAGGCTGGAAGAGAG	
qBTV NS3-F	AAGAGCTGAGTCTGGTCCGT	qRT-PCR
qBTV NS3-R	AAGTGCAACAGTAGGCATCG	
si-oISG15-1	CCAUGAUGGUAUCCGAGCUTT	Knockdown of oISG15
si-oISG15-2	UCCUGCUGAUGGUGCAGAATT	
si-oISG15-3	GCACCGUGUUCAUGAAUCUTT	

To investigate the characteristics of the oISG15 gene, specific primers were designed according to the predicted mRNA sequence of oISG15 (NM_001009735). Total RNA was extracted from MDOK cells infected with BTV-1 at an MOI of 1 for 48 h. The RT-PCR was performed with the One-step PrimeScript RT reagent kit (TaKaRa), and the PCR products were ligated into the pMD-18T Vector (TaKaRa) and verified by DNA sequencing. The comparison of ISG15 amino acid sequences between ovine and other species (the sequences were obtained from NCBI) was performed by *BioEdit* multiple sequence alignment.

### 2.3. Plasmids and siRNA

The CDS of oISG15 was subcloned into the pCAGGS-HA vector, and the recombinant plasmid was named as oISG15-HA. The “LRLRGG” motif is responsible for ISGylation, and the substitution of the last two glycines to alanines abolishes its conjugation activity. The conjugation-defective mutant plasmid mut-oISG15-HA was constructed by PCR mutagenesis.

A series of recombinant plasmids expressing viral proteins that contain His and Myc tag was constructed as described previously (Kang et al., 2022). The Flag-tagged wild type ubiquitin expressing plasmid (Ub-Flag) and other seven site-directed mutant plasmids (K6, K11, K27, K29, K33, K48, and K63) were preserved in our lab. Three pairs of short interfering RNAs (si-oISG15-1, si-oISG15-2, and si-oISG15-3) and a negative control (NC) were designed and synthesized to knockdown the endogenous oISG15. The primers and siRNA sequences are presented in Table 1.

### 2.4. Analysis of BTV replication

To evaluate the effect of oISG15 on BTV replication, the MDOK cells were infected with BTV-1 following oISG15 overexpression or knockdown. The BTV NS3 RNA was measured by qRT-PCR, and the BTV viral proteins and virus titers were determined by Western blot and plaque formation assay, respectively. For the ISG15 overexpression assay, the MDOK cells were transfected with oISG15-HA using Lipofectamine™ 3000 following the manufacturer’s instructions (Invitrogen). For the knockdown assay, three pairs of siRNA were transfected into MDOK cells. At 24 h post-transfection, the cells were infected with BTV-1 at an MOI of 1, and the culture supernatants were collected to measure the virus titers. The cells were washed with PBS and the lysates were harvested with cell scraps to measure the viral RNA and protein levels at 12, 24, and 48 hpi.

#### 2.4.1. Quantitative real-time PCR

Total RNA was extracted from the cell samples mentioned above using TRIzol reagent. Then, the first-strand cDNA was prepared using the PrimeScript™ RT reagent kit (TaKaRa). Quantitative real-time PCR was performed using SYBR Green Mix (TaKaRa) and the specific primers for BTV NS3 gene. The procedure was carried out with incubation at 95°C for 30 s, followed by 40 cycles of 5 s at 95°C, and 30 s at 60°C. β-actin was used to normalize the total RNA. The relative levels of NS3 RNA were calculated using the 2^–ΔΔCT^ method.

#### 2.4.2. Western blot

The cell lysates from collected samples were separated on an SDS-polyacrylamide gel, and then transferred onto polyvinylidene difluoride (PVDF) membranes (Millipore). The expression of oISG15 and mut-oISG15 were detected by mouse anti-HA tag antibody (ProteinTech, 51064-2-AP, 1:4,000). The viral proteins were detected by rabbit anti-VP6 (dilution = 1:1,000) and rabbit anti-NS1 antibody (dilution = 1:1,000), and both antibodies were prepared in our lab.

The HEK-293T cells were co-transfected with viral recombinant plasmids expressing VP3, VP4, VP5, NS1 protein, and oISG15-HA plasmid, respectively. Viral proteins were detected by mouse anti-Myc tag antibody (Cell Signaling Technology, 20229, 1:1,000), the ubiquitin conjugation was tested using anti-Flag antibody (ProteinTech, 66008-4-Ig, 1:5,000), and the oISG15 was detected by mouse anti-HA tag antibody (ProteinTech, 51064-2-AP, 1:5,000).

All the target proteins were visualized using a ChemiDoc MP imaging system (Bio-Rad) after incubation with the corresponding secondary antibodies (Abcam, ab6789/ab6721, 1:10,000). The band intensities of the viral proteins were measured using ImageJ software and normalized to that of β-actin.

#### 2.4.3. Plaque formation assay

The supernatant from the MDOK cells transfected and then infected with BTV-1 was clarified by centrifugation at 4°C. The serially diluted supernatant was inoculated into BHK-21 monolayers grown in 24-well culture plates for 1 h at room temperature (RT) on a shaker. The inoculum was replaced with a cell culture medium containing 1.5% low-melting agarose (Invitrogen), and then maintained at 37°C for 48–72 h. The plaques were visualized by crystal violet staining. Viral titers were calculated using the number of PFUs per milliliter.

### 2.5. Co-immunoprecipitation

The monolayer cells of HEK-293T were transiently co-transfected with various viral protein-expressing plasmids and oISG15-HA, respectively. The cells were harvested at 24 h post-transfection, and washed with cold PBS, then lysed using NP-40 buffer (Beyotime) [50 mM Tris (pH 7.4), 150 mM NaCl, 1% NP-40] with protease inhibitors for 30 min. After verifying the co-expression of target proteins, the lysates were immunoprecipitated with anti-Myc or anti-HA antibody overnight at 4°C. The beads bounded with protein A and protein G (Sigma-Aldrich) were added and gently shaken for 4 h at 4°C. Then, the antibody–protein complexes were washed by slow centrifugation in NP-40 buffer five times. The beads were resuspended and boiled for 5 min in loading buffer and analyzed by Western blot.

### 2.6. Confocal analysis

MDOK cells grown on the coverslips were transfected with BTV protein-expressing plasmids and oISG15-HA, respectively. After incubation for 24 h, the transfected cells were fixed with 4% paraformaldehyde for 15 min and permeabilized with 0.5% Triton X-100 for 10 min at RT. The samples were blocked with 3% bovine serum albumin (BSA) solution and incubated with the primary antibody, which was diluted in a 1% BSA solution. The HA-tagged oISG15 was detected by rabbit or mouse anti-HA antibody, and the Myc-tagged BTV protein was detected by mouse or rabbit anti-Myc antibody. After incubation for 12 h at 4°C, either Alexa Fluor-conjugated goat anti-rabbit (Abcam, ab150077/ab175471, 1:1,000) or goat anti-mouse secondary antibody (Abcam, ab150113, 1:1,000) was added. Then, the samples were gently shaken for 1 h and stained with Hoechst 33342 (Sigma-Aldrich) for 10 min at RT. Images were captured using a confocal microscope.

### 2.7. Chemical inhibitor assay

Cycloheximide (CHX, Aladdin), a eukaryotic translation inhibitor, was used to investigate the effect of oISG15 on the stability of BTV proteins. CHX was dissolved in DMSO (50 μg/ml) and added to the culture medium, which co-transfected with BTV protein-expressing plasmids VP4 and NS1, respectively, and oISG15-HA. The cell samples were harvested at indicated timepoints, and the expression levels of BTV proteins were determined by Western blot. To investigate the degradation mechanism of the NS1 and VP4 proteins in host cells, the MDOK cells transfected with NS1 or VP4 expressing plasmid were then treated with the proteasome inhibitor MG132 (Merck, 10 μM), caspase inhibitor A-VAD-FMK (Sigma, 20 μM), or lysosomal inhibitor Chloroquine (CQ, Sigma, 50 μM). The cells were collected and subjected to Western blot assays.

## 3. Results

### 3.1. BTV infection upregulated the transcription level of the oISG15 gene

**FIGURE 1:**
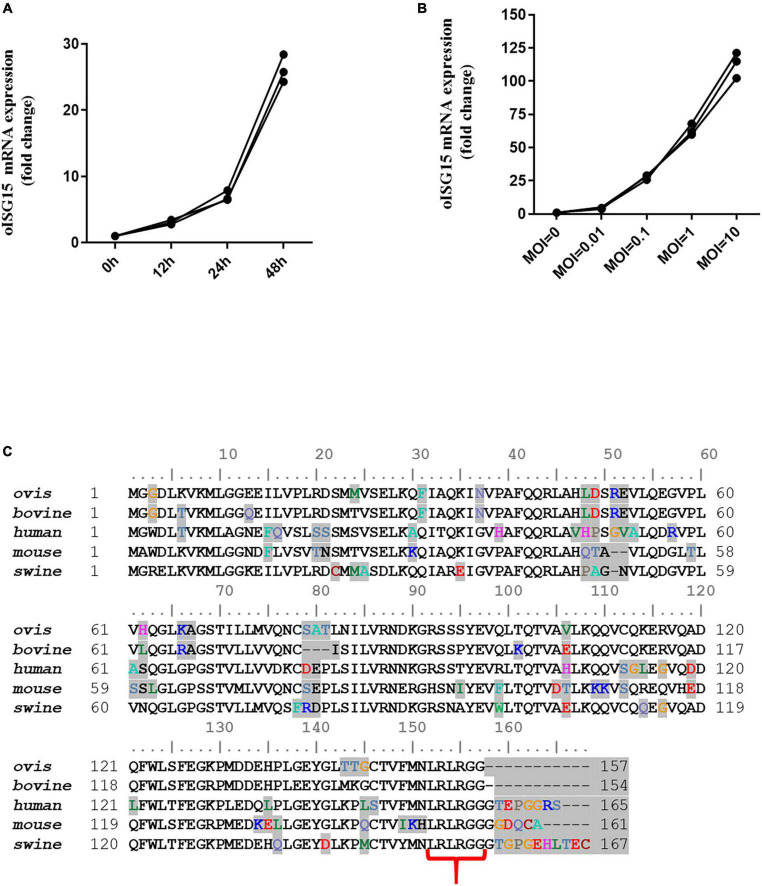
Bluetongue virus infection induced the transcription of oISG15. **(A)** MDOK cells were infected with BTV-1 at an MOI of 0.1. At 12, 24, and 48 hpi, the BTV-infected cells were harvested to quantify the relative levels of ISG15 mRNA using qRT-PCR. **(B)** MDOK cells were infected with BTV-1 at MOIs of 0, 0.01, 0.1, and 1. The mRNA levels of oISG15 were quantified by qRT-PCR at 48 hpi. The relative fold changes were determined by the 2^–ΔΔCT^ method. The three values were triplicates for each sample. **(C)** Sequence alignment of mammalian ISG15. Different amino acids are shaded in gray. The LRLRGG motifs of these sequences are indicated by red brackets.

Total RNA was extracted from BTV-infected MDOK cells, and the mRNA level of oISG15 was determined by qRT-PCR. The results indicated that the transcription of oISG15 increased by 2. 1-, 6. 0-, and 25.2-fold in BTV-infected cells at 12, 24, and 48 hpi, respectively, compared to uninfected cells (Figure 1A). The oISG15 mRNA level also exhibited a BTV MOI-dependent manner, with a 3. 5-, 26. 8-, 62. 2-, and 113.9-fold increase in BTV-infected cells with MOIs of 0.01, 0.1, 1, and 10, respectively, compared with the control group (Figure 1B). The CDS of the oISG15 gene was obtained by RT-PCR and sequenced. A comparison of the amino acid sequences of ISG15 with that of other species showed that the bovine and ovine ISG15 contained the motif of “LRLRGG” at the C-termini. However, the C-termini of human, mouse, and porcine ISG15 contained 5–8 amino acids following LRLRGG motif (Figure 1C).

### 3.2. oISG15 enhanced BTV replication in an ISGylation-independent manner

**FIGURE 2:**
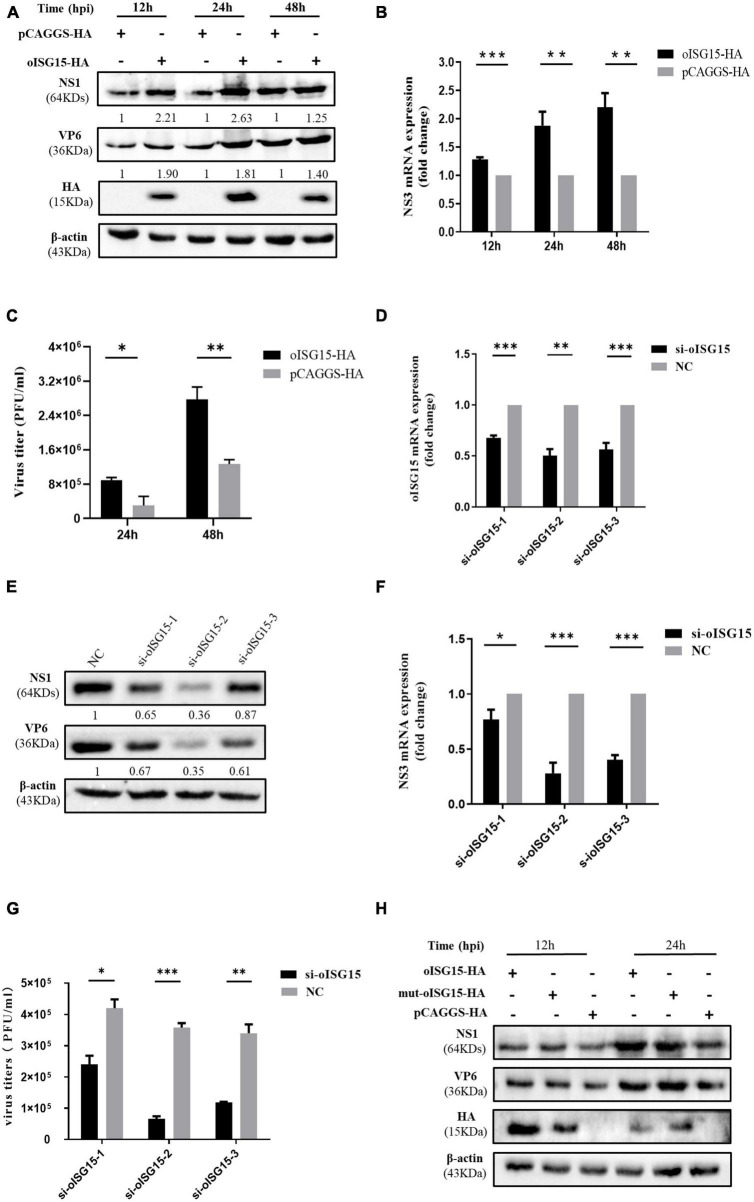
Ovine ISG15 upregulated BTV replication in an ISGylation-independent manner. **(A)** The expression abundance of NS1 and VP6 was detected by Western blot at 12, 24, and 48 hpi after transfecting the MDOK cells with 1.6 μg of oISG15-HA or pCAGGS-HA and then infecting with BTV at an MOI of 1. The expression of NS1 and VP6 was normalized to the β-actin. Then, the relative intensity to the control group at different time points was measured by ImageJ and marked below the bands. **(B)** The relative fold change of NS3 RNA level was measured by qRT-PCR at 12, 24, and 48 hpi after oISG15 overexpression and BTV infection. β-actin was used as a housekeeping gene to normalize the target gene. **(C)** The culture supernatant of MDOK cells was collected following oISG15-HA or pCAGGS-HA transfection and BTV-1 infection at an MOI of 1. Virus titers at 24 and 48 hpi were determined with plaque formation assay. **(D)** MDOK cells were transfected with 60 pmol of si-oISG15-1, si-oISG15-2, si-oISG15-3, or NC, and the knockdown efficiency of each siRNA was determined by qRT-PCR for 48 h. **(E)** The expression of BTV-1 NS1 and VP6 proteins was evaluated after the knockdown of endogenous oISG15 by three pairs of siRNA and then BTV-1 infection at 24 hpi. The expression abundance of NS1 and VP6 relative to the NC transfected group was measured by ImageJ and normalized to the β-actin, as indicated below the bands. **(F)** The fold change of BTV-1 NS3 RNA in MDOK cells was quantified using qRT-PCR after siRNA transfection and then BTV-1 infection at an MOI of 1. **(G)** A plaque assay was used to determine the viral titer in MDOK cells in which endogenous oISG15 was knocked down or not. **(H)** 1.6 μg of oISG15-HA, mut-oISG15-HA, and pCAGGS-HA was transfected into MDOK cells, respectively, and then infected with BTV-1. The cells were harvested at 12 and 24 hpi, and the NS1 and VP6 proteins were detected by Western blot. Differences between the oISG15 overexpression or knockdown and the control groups are denoted with asterisks to indicate significance levels: *P < 0.05, ^**^P < 0.01, and ^***^P < 0.001. Error bars represent the mean ± SD of the data from three independent experiments.

To evaluate the effect of oISG15 on BTV replication, the production of viral protein, viral RNA, and virus titer was measured in MDOK cells with oISG15 overexpression or knockdown. oISG15-HA or pCAGGS-HA was transfected into MDOK cells and then infected with BTV-1 at an MOI of 1. As shown in Figure 2A, the expression of BTV NS1 and VP6 was significantly enhanced in oISG15-overexpressing cells at different timepoints. Meanwhile, the BTV NS3 RNA level in oISG15-HA transfected cells was upregulated by an average of 1. 28-, 1. 88-, and 2.21-fold at 12, 24, and 48 hpi, compared with vector-transfected group (Figure 2B). The virus titers in the supernatant of oISG15-overexpressing cells were 1.96- and 1.19-fold greater than that non-expressing cells at 24 and 48 hpi, respectively (Figure 2C).

To determine whether the endogenous oISG15 contributes to BTV replication, three pairs of siRNA were designed to target the oISG15 mRNA (Table 1). The knockdown efficiency of si-oISG15-1, si-oISG15-2, and si-oISG15-3, was 32, 50, and 44%, respectively (Figure 2D). Correspondingly, the knockdown of oISG15 considerably suppressed BTV infection. Compared with NC siRNA transfected cells, the expression of NS1 protein in si-oISG15-1, si-oISG15-2, and si-oISG15-3 transfected cells reduced by 35, 64, and 13%, respectively, at 24 hpi. The expression of VP6 protein was 39–65% lower than that of the NC group (Figure 2E). qRT-PCR results indicated that the NS3 RNA level reduced by 23, 72, and 60% in the oISG15 knockdown cells, compared to the NC group (Figure 2F). Furthermore, the released virus titer in si-oISG15-1, si- oISG15-2, and si-oISG15-3 transfected cells showed a 0. 43-, 0. 82-, and 0.66-fold decrease, compared with NC transfected group, respectively (Figure 2G). These data demonstrated that oISG15 enhanced BTV replication.

To examine whether the upregulated effect of oISG15 on BTV replication is dependent on ISGylation, the “LRLRGG” motif of oISG15-HA, which is critical for posttranslational modification, was substituted by “LRLRAA” and transfected into MDOK cells, and then infected with BTV-1. We found that the expression of viral proteins was not significantly different in oISG15-HA and mut-oISG15-HA transfected cells (Figure 2H). Therefore, we concluded that the regulation of viral replication by oISG15 is independent of ISGylation.

### 3.3. oISG15 upregulated the expression of VP3, VP4, VP5, and NS1

**FIGURE 3:**
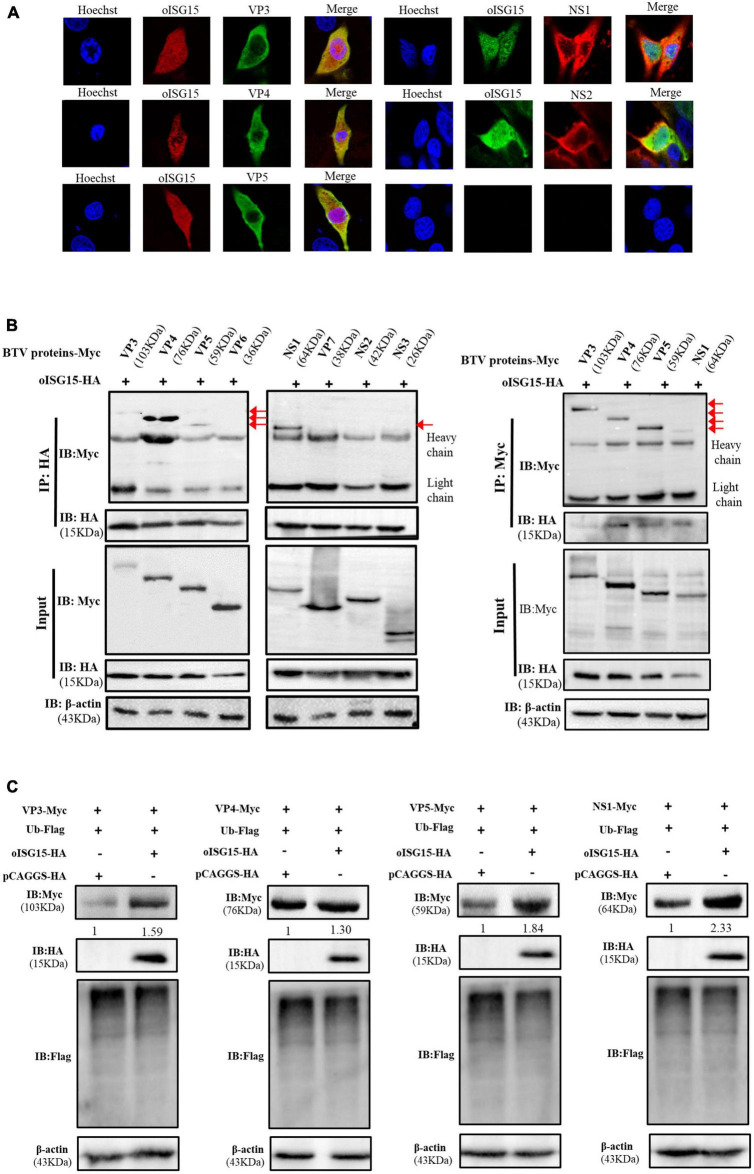
Ovine ISG15 upregulated the expression abundance of VP3, VP4, VP5, and NS1. **(A)** MDOK cells were co-transfected with Myc-tagged viral protein plasmids and oISG15-HA, respectively, and incubated for 24 h. BTV VP3–VP7 were detected by mouse anti-Myc antibody and NS1–NS3/NS3A were detected by rabbit anti-Myc antibody. The oISG15 was detected by rabbit or mouse anti-HA antibody and nuclei were stained by Hoechst 33342 (blue). A confocal assay was used to analyze the co-localization between BTV proteins and oISG15. The magnification of confocal microscope is 100×. **(B)** VP3–VP7 and NS1–NS3/NS3A expressing plasmids were co-transfected with oISG15-HA into HEK-293T cells for 48 h. The cells were incubated with the indicated antibodies to detect the co-expression of the target proteins. Co-IP assays were performed to determine the interaction between BTV proteins and oISG15. **(C)** The VP3, VP4, VP5, or NS1 expressing plasmid was co-transfected with Ub-Flag and oISG15-HA. After 48 h, the expression of oISG15, ubiquitin, and four BTV proteins were detected by Western blot. The relative intensity to the pCAGGS-HA transfected group was measured by ImageJ and marked below the bands.

To clarify the mechanism by which oISG15 positively regulates BTV replication, we analyzed the interaction of oISG15 and BTV proteins using confocal microscopy and co-immunoprecipitation (Co-IP). The viral protein-expressing plasmids (VP3–VP7, NS1–NS3, and NS3A) and oISG15-HA were co-transfected into MDOK cells. The results showed that oISG15 co-localized with BTV VP3, VP4, VP5, NS1, and NS2 (Figure 3A). To verify the interaction of BTV proteins with oISG15, the plasmids was co-transfected into HEK-293T cells, and Myc-tagged BTV proteins were detected with anti-Myc tag antibody after precipitation with anti-HA tag antibody. As shown in Figure 3B, VP3, VP4, VP5, and NS1 were precipitated by anti-HA-tag antibody, whereas the other viral proteins, especially NS2, were not. A reverse immunoprecipitation experiment also indicated that these four BTV proteins interacted with oISG15.

The expression levels of oISG15-interacting viral proteins were measured under the condition of ubiquitin overexpression. The results showed that the expression of VP3, VP4, VP5, and NS1 increased 1. 59-, 1. 3-, 1. 84-, and 2.33-fold, respectively, compared with the control group. The ubiquitination level also decreased (Figure 3C).

### 3.4. oISG15 enhanced the stability of VP4 and NS1

**FIGURE 4:**
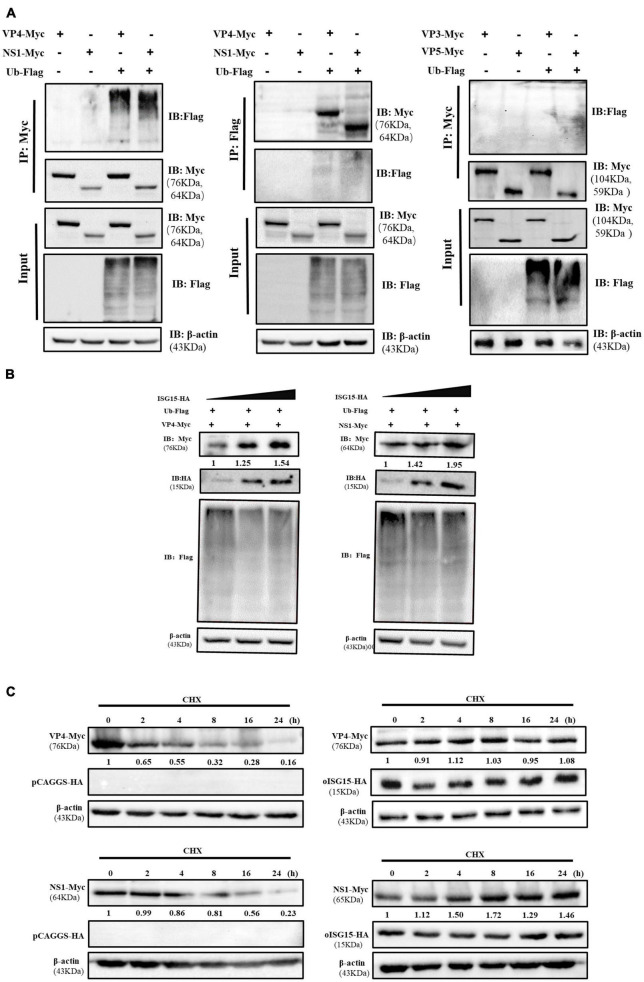
Ovine ISG15 enhanced the expression and stability of VP4 and NS1. **(A)** The cell lysate was incubated with the corresponding antibodies following the co-transfection of Myc-tagged VP3, VP4, VP5, and NS1 expressing plasmids with Flag-tagged ubiquitin plasmid in HEK-293T cells. The ubiquitination bands were determined by Western blot, and the bands of VP4 and NS1 were detected in a reverse Co-IP assay. **(B)** The overexpression of oISG15 promoted the abundance of VP4 and NS1 in a dose-dependent manner. A gradually increasing dose (0.5, 1, and 1.5 μg) of oISG15-HA was co-transfected with 1 μg of VP4 or NS1 expressing plasmids into HEK-293T cells, and after 24 h incubation, VP4, NS1, and ubiquitination were detected by Western blot. The relative intensity of VP4 and NS1 to the oISG15-HA transfected group was normalized with the corresponding β-actin and marked below the bands. **(C)** oISG15-HA or pCAGGS-HA was co-transfected with VP4 and NS1 expressing plasmid for 12 h, and cell lysates were harvested at 0, 2, 4, 8, 16, and 24 h after CHX treatment. The expression of viral proteins was detected by Western blot and the intensity was measured by ImageJ. The relative intensity to CHX treatment at 0 h was marked below the bands.

To exam whether the BTV proteins were associated with ubiquitin, the VP3, VP4, VP5, or NS1 expressing plasmid was co-transfected with Flag-Ub plasmid, which expresses Flag-tagged ubiquitin, into HEK-293T cells. The immunoprecipitation and the reverse experiment indicated that VP4 and NS1 interacted with ubiquitin, but that VP3 and VP5 did not (Figure 4A). In addition, the expression abundance of VP4 and NS1 increased in an oISG15-dependent manner, but the level of polyubiquitination was negatively correlated with the expression of oISG15 (Figure 4B). The Western blot results indicated that VP4 and NS1 expression in oISG15-HA-transfected cells were not significantly reduced at 2, 4, 8, 16, and 24 h after CHX treatment, but decreased in a time-dependent manner without oISG15 overexpression (Figure 4C). These results suggested that oISG15 enhanced the expression abundance and stability of VP4 and NS1 proteins.

### 3.5. oISG15 disturbed the degradation of NS1 protein

**FIGURE 5:**
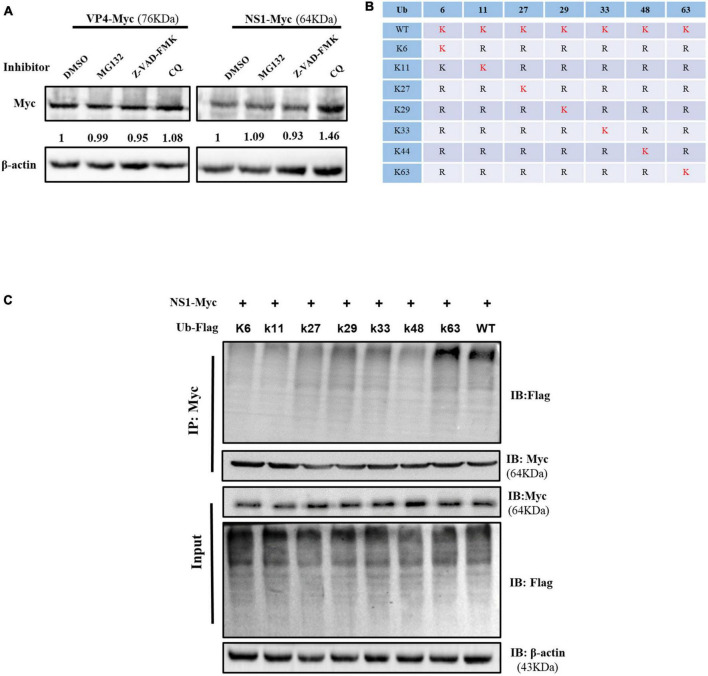
Ovine ISG15 interfered with NS1 degradation. **(A)** MDOK cells with overexpressed VP6 and NS1 were treated with MG132, Z-VAD-FMK, CQ, and DMSO (control), respectively. Viral proteins were detected by Western blot, and the relative expression to the control group was normalized to the β-actin. **(B)** Schematic representation of seven ubiquitin mutants (K6, K11, K27, K29, K33, K48, and K63). **(C)** Ubiquitin and ubiquitin-mutant plasmids were co-transfected with NS1 expressing plasmid for 24 h. The cell lysates were incubated with anti-Myc antibodies for a Co-IP assay and detected by Western blot.

The inhibitors of proteasome, caspase, and autophagy were used to investigate the pathway involved in VP4 and NS1 degradation. Compared with the DMSO-treated group, the expression level of NS1 in the MG-132 and Z-VAD-FMK treatment groups did not show significant changes, while that in the CQ treatment group increased, suggesting that NS1 was degraded through the autophagy pathway (Figure 5A). The expression of VP4 showed no obvious differences between the inhibitor-treated cells and untreated cells.

To further explore the type of polyubiquitination chains bound to NS1, Flag-Ub WT and seven mutant plasmids (Figure 5B) were co-transfected with NS1 expressing plasmid into HEK-293T cells, respectively. The Co-IP assay showed that the NS1 modification was reverted by the K63-linked polyubiquitin chain formation, suggesting that oISG15 enhanced the stability of NS1 by interfering with the autophagy pathway (Figure 5C).

## 4. Discussion

Interferon-stimulated gene 15 was found to play important roles in the immune regulation of virus infection. Here, we first demonstrated that the transcription level of oISG15 increases significantly with BTV infection in an MOI- and time-dependent manner. The overexpression of oISG15 facilitates BTV replication, and the knockdown of oISG15 inhibits virus replication. ISG15 is a ubiquitin-like protein that is conjugated to intracellular proteins via the LRLRGG motif. We found that this motif was not related to BTV replication. In addition, the ubiquitination level decreased with the overexpression of oISG15, enhancing the stability of VP4 and NS1. Inhibitor treatment and K63-linked polyubiquitin experiments suggested that NS1 degraded through the autophagy pathway. We thus conclude that oISG15 promotes BTV replication by increasing the stability of viral proteins.

Interferon-stimulated gene 15 has two ubiquitin-like domains that contain four β-sheets and a single α-helix. These two domains are connected by a polypeptide sequence described as a “hinge” (Narasimhan et al., 2005). ISG15 of multiple species ultimately terminate with the same LRLRGG sequence. Nevertheless, ISG15 in fish, bovine, and ovine lacks the short extension following the LRLRGG motif before the cleavage of the precursor via protease (Ritchie and Zhang, 2004; Zhang and Zhang, 2011). As a ubiquitin-like protein, ISG15 differs from ubiquitin in terms of its highly conservative properties. Some sequence identities of ISG15 among different species are even less than 40% (Dzimianski et al., 2019). The sequence diversity may respond to the high sequence variability and the cross-species compatibility of ISG15 enzymes.

The IFN-inducible protein ISG15 and ISGylation have been reported as antiviral factors in multiple DNA and RNA viral infections. For example, the ISGylation of the Influenza A virus (IAV) NS1 protein inhibited nuclear translation and contributed to the antiviral response of IFN-β (Zhao et al., 2010). ISG15 modification of the Cytomegalovirus (HCMV) pUL26 protein suppressed its activities to promote viral growth and inhibit the IFN signal pathway (Kim et al., 2016). The free intracellular ISG15 interacted with the ubiquitin ligase-Nedd4, which inhibits the ubiquitination of the VP40 protein and results in the disruption of Ebola VP40 VLP release (Okumura et al., 2008). In contrast, our study demonstrated that oISG15 acted as a pro-viral protein and enhanced BTV replication. Similar results were also observed by Speer et al. (2016), who found that the proliferation levels of herpes simplex virus I (HSV-I), HCMV and IAV were significantly lower in IFN-α/β-primed ISG15-deficient fibroblasts than in control cells. This means that human ISG15 attenuates protection against viruses in individuals (Speer et al., 2016). The overexpression of ISG15 also promoted Zika virus infection, not by increasing virus replication directly, but by downregulating the JAK/STAT signaling pathway and reduced the production of ISGs (Wang et al., 2020). Bawono et al. (2021) found that the silencing of ISG15 significantly restrained hepatitis B virus (HBV) infection, resulting in a 30% reduction of HBV rcDNA. The silencing of USP18 (an ISG15 de-conjugating enzyme) increased HBV replication in Hep38.7-Tet cells (Bawono et al., 2021). These results indicated that the ISGylation of host molecular or viral protein participated in the resistance to IFN-I-mediated antiviral activity. However, here we showed that the upregulation of BTV replication by oISG15 was not dependent on ISGylation. Similarly, free intracellular ISG15 lead to a decrease in sensibility to HIV-1 infection because the interaction between ISG15 and ISGylation deconjugating enzyme USP18 negatively regulated the IFN response (Speer et al., 2016). Other reports showed that free ISG15-deficient enhanced type I IFN activity and persistent phosphorylation of STAT1 and STAT2, which enhanced the production of ISG proteins and inhibited viral replication (Dos Santos and Mansur, 2017).

To further study the mechanism by which oISG15 promotes BTV replication, a Co-IP assay was performed to explore which protein interacts with ISG15. Four viral proteins were found to interact with ISG15, and the overexpression of ISG15 improved the expression abundance of these viral proteins. Similarly, Minami et al. (2017) reported that unconjugated ISG15 was involved in HCV replication, by colocalizing with HCV NS5A protein and interacting with NS5A in an ISGylation-independent manner. Further data showed that the NS5A protein was modified by ISG15, and the ISGylation promoted the recruitment of the CypA protein, facilitating HCV replication (Abe et al., 2020). In addition, it has been reported that there is antagonistic relationship between ISG15 and ubiquitination. Free intracellular ISG15 competes with the E2 ligase enzyme with ubiquitin. An increase of ISG15 caused a decrease in polyubiquitinated proteins, and cellular protein degradation was often related to ubiquitination modification (Malakhova and Zhang, 2008; Song and Luo, 2019). The ubiquitin itself was a substrate of ISG15, and the binding of the two proteins could form a ubiquitin-ISG15 mixed chain that negatively regulated the level of ubiquitination in cells (Fan et al., 2015). Therefore, we investigated the interaction between these four viral proteins and ubiquitin to determine whether ISG15 influenced the ubiquitin-dependent degradation of viral protein. Only VP4 and NS1 were found to be associated with ubiquitin. These results implied that oISG15 expression promoted the stability of these two viral proteins. In addition, the NS1 can be modified by the K63-linked ubiquitination chain, and the degradation of NS1 may be involved in the autophagy pathway. As the second most abundant ubiquitylation, K63-linked ubiquitin often involves signal transduction and DNA repair. It is also a response to the modification of membrane proteins, which is essential for multivesicular bodies and subsequent lysosomal degradation in mammals (Erpapazoglou et al., 2014; Fan et al., 2015). It is noteworthy that NS1 is the earliest and most expressed protein during BTV infection, and it upregulates the transcription and translation levels of BTV RNA (Kerviel et al., 2019). Therefore, our results provided evidence for the degradation mechanism of NS1 through autophagy pathway. Ovine ISG15 might disturb K63-linked ubiquitination to promote BTV replication.

## 5. Conclusion

In summary, we observed a significant increase in oISG15 mRNA in BTV-infected MDOK cells. The overexpression of free intracellular oISG15, rather than ISGylation enzyme system, promoted BTV replication, and the knockdown of endogenous oISG15 inhibited BTV replication. The Co-IP assay showed that four viral proteins interacted with oISG15, and the overexpression of oISG15 enhanced the abundance of viral proteins. The data indicated that oISG15 overexpression improved the stability of VP4 and NS1. oISG15 might reduce the degradation of NS1 protein by disturbing the autophagy pathway. This study provides new insights into the interaction between oISG15 and BTV replication. The results will be useful for our understanding of the ISG15 function during viral infection and may contribute to exploring novel candidates for anti-BTV infection.

## Data availability statement

The datasets presented in this study can be found in online repositories. The names of the repository/repositories and accession number(s) can be found in the article/supplementary material.

## Ethics statement

Ethical approval was not required for the studies on animals in accordance with the local legislation and institutional requirements because only commercially available established cell lines were used.

## Author contributions

JD and HY conceived the project and provided critical revisions of the manuscript. DK and GZ carried out the experiments and drafted the manuscript. SG and ZT designed the study, analyzed the data, and provided critical revisions of the manuscript. GG, GL, and JL collected the data and provided critical revisions of the manuscript. All authors contributed to the article and approved the submitted version.
